# The effect of Oleoylethanolamide supplementation on lipid profile, fasting blood sugar and dietary habits in obese people: a randomized double-blind placebo-control trial

**DOI:** 10.1186/s12902-024-01738-7

**Published:** 2024-10-08

**Authors:** Alireza Ostadrahimi, Yaser Khajebishak, Fardin Moradi, Laleh Payahoo

**Affiliations:** 1 Nutrition Research Center, Department of Clinical Nutrition, School of Nutrition & Food Sciences, Tabriz University of Medical Sciences, Tabriz, Iran; 2 Nutrition Sciences, Department of Nutrition, Maragheh University of Medical Sciences, Maragheh, Iran; 3 Student Research Committee, School of Nutritional Sciences and Food Technology, Kermanshah University of Medical Sciences, Kermanshah, Iran; 4 Nutrition Sciences, Medicinal Plants Research Center, Maragheh University of Medical Sciences, Maragheh, Iran

**Keywords:** Obesity, Oleoylethanolamide, PPAR alpha, Lipids, Blood glucose, Feeding Behavior

## Abstract

**Background:**

Abnormalities in biochemical parameters and changes in eating habits are considered complications of obesity. Oleoylethanolamide (OEA), an endocannabinoid-like compound, has been shown to have protective effects on many metabolic disorders. Given this evidence, the present study aimed to assess the effects of OEA on lipid profile parameters, fasting blood sugar (FBS), and dietary habits in healthy obese people.

**Methods:**

In this randomized, double-blind, placebo-controlled clinical trial, which was carried out in 2016 in Tabriz, Iran, 60 obese people were enrolled in the study based on inclusion criteria. The intervention group consumed 125 mg of OEA capsules, and the placebo group received the same amount of starch twice for 8 weeks. Blood samples (5 mL) were taken at baseline and the end of the study in a fasting state. Serum concentrations of FBS, triglycerides (TGs), high-density lipoprotein cholesterol (HDL-C), and total cholesterol (TC) were measured by enzymatic methods using commercial kits. The low-density lipoprotein cholesterol (LDL-C) concentration was obtained using the Friede-Wald formula. To assess dietary habits, a food frequency questionnaire (147 items) was used at baseline and the end of the study. A value less than < 0.05 was considered to indicate statistical significance.

**Results:**

The TG concentration decreased significantly in the intervention group (mean (SD): 166.29 (70.01) mg/dL to 142.22 (48.05) mg/dL, *p* = 0.047). Changes in the placebo group were not significant (*p* > 0.05). After adjusting for baseline values and demographic characteristics, the difference in TG between groups remained significant (*p* = 0.044). Changes in other biochemical parameters were not significant. There was no significant difference between or within groups in terms of food groups.

**Conclusion:**

OEA, as a complementary agent, plays a protective role in TG regulation. However, future studies with longer durations are needed to explore the impact of OEA on regulating dietary habits and to identify the mechanisms related to metabolic abnormalities in obese people.

**Trial Registration:**

The study was registered in the Iranian Registry of Clinical Trials (IRCT) center as IRCT201607132017N30 with URL. www.IRCT.IR in date 03/10/2016.

## Background

Nowadays, the rate of non-communicable has increased dramatically due to unhealthy eating, sedentary lifestyles, and low levels of physical activity [1, 2]. Obesity, a metabolic disorder of fat accumulation, appears to be caused by an imbalance between energy intake and energy expenditure [3, 4]. It is considered a crucial risk factor for non-communicable diseases such as cancer, diabetes, and cardiovascular diseases. More than 70% of premature disability and mortality worldwide are attributed to non-communicable diseases [5]. In addition to impaired health, other consequences of obesity are reduced reproduction, social disadvantages, reduced quality of life, and unemployment [6]. According to the WHO report, 14% of men and 18% of women in 2020 were obese worldwide, and this figure is estimated to increase by 23% and 27%, respectively, by 2035 [7]. Globally, in Iran, the prevalence of obesity was 13.44%, and the overall prevalence of obesity and overweight has been reported to be nearly 35.1% [8]. Thus, applying effective approaches to control obesity and related comorbidities seems to be a priority in healthcare systems.

The main manifestations, especially abdominal obesity, are dyslipidemia, insulin resistance, and inflammation [9–11]. A growing body of evidence indicates that obese people who experience metabolic dyslipidemia have a high level of triglycerides and a low level of high-density lipoprotein (HDL-C) [12, 13]. Dyslipidemia is a widely accepted risk factor for coronary artery disease and is an important feature of metabolic syndrome [14].

In addition to genetics, environmental factors are involved in the incidence of obesity [15, 16]. Obese people mostly adhere to unhealthy dietary habits and sedentary lifestyles [17]. Various approaches, such as surgery, drug therapy, appetite suppressants, and lifestyle modifications, are used for weight loss and treatment of abnormalities in obese people [18].

Recently, applying natural components has attracted more attention as a useful solution in the management of obesity due to low side effects and more effectiveness [10, 19]. One of the potential components that have recently attracted increased interest in modifying obesity-related disturbances is oleoylethanolamide (OEA). OEA is expressed in tissues such as neurons, the small intestine, and adipose tissue and acts as an endogenous ethanolamide fatty acid [20]. Dietary sources of OEA are cocoa powder, nuts, and oatmeal, and the amount of OEA in these foods is less than 2 µg/g [19]. Accumulating evidence has confirmed the protective role of OEA in inflammation and neurodegenerative diseases, such as Parkinson’s disease, pain relief, apoptosis induction, weight loss, lipolysis stimulation, and fatty acid oxidation enhancement [19–23]. Promising evidence also suggests that OEA improves biochemical abnormalities such as dyslipidemia in chronic diseases via the induction of the expression of genes involved in energy hemostasis and feeding behavior and the regulation of lipid metabolism, such as GPR119 and PPAR-α [24–27]. Tutunchi et al. [28] showed that the supplementation of 76 obese people with NAFLD with 250 mg OEA for 12 weeks significantly decreased TG, dietary intake, appetite, and FBS and increased HDL-C. In another study, Li et al. [29] reported the administration of 5 mg/kg/day OEA to rats for 6 or 17 weeks. The results showed that OEA decreased TG and TC significantly at the end of the intervention period. The satiety-sensing ability of OEA has been discussed in previous studies [30, 31].

Given the promising evidence of OEA’s effectiveness in improving biochemical markers and inflammation due to its natural components with low side effects, we assumed that OEA can be effective in improving lipid profiles, FBS, and eating-related habits. Given the growing prevalence of obesity in recent decades the importance of discovering natural approaches in the control of obesity and the limited human studies that have assessed the role of OEA on abnormalities observed in obese people including lipid profiles, FBS, and dietary habits, the present study aimed to assess the effects of OEA on the FBS, lipid profile and dietary habits in healthy obese people.

## Methods

This randomized, double-blind, placebo-controlled trial was conducted on 60 healthy obese people who were referred to the healthcare centers of Tabriz University of Medical Sciences, Iran, in 2016. Individuals aged between 18 and 59 years with a BMI of 30 kg/m^2^ and above were considered as inclusion criteria. The exclusion criteria were people who suffered from kidney or liver disease, heart failure, or rheumatic or gastrointestinal disease; who were pregnant, menopausal, or breastfeeding; or who were taking synbiotic, omega 3, multivitamin, or mineral supplements or weight-lowering drugs during the past month. This study was carried out as a part of a megaproject and the results of other variables including PPAR-alpha gene expression, inflammation and oxidative stress, appetites, *Akkermansia muciniphila* abundance changes, dietary intake, and anthropometric measurements were published previously [19, 20, 32]. The protocol of the study was approved by the ethics committee of Tabriz University of Medical Sciences with registration number IR.TBMED.REC.1395.618 in date 2016/10/03. The study was registered in the Iranian Registry of Clinical Trials (IRCT) center as IRCT201607132017N30 with URL: www.IRCT.IR.

After participants provided an informed written consent form, a demographic questionnaire was completed by the participants. The participants were randomly (using the random allocation software (RAS) method) divided into an intervention group (two capsules containing 125 mg of OEA daily) and a control group (the same amount of starch) and followed for eight weeks.

It was emphasized to take supplements before lunch and dinner. The synthesis of the supplement was described in detail in a previous study [20]. The appearance and color of the supplements were similar in both groups. The main investigator and the participants were blinded until the end of the study. All participants were advised no change their usual dietary intake and physical activity level during the follow-up period. The physical activity of participants was assessed using the short form of the International Physical Activity Questionnaire (IPAC) questionnaire at baseline and at the end of study.

Body weights of eligible subjects were measured with light clothing using the Seca scale (Seca, Hamburg, Germany), and heights were measured with a stadiometer (Seca) while fasting at baseline. Body mass index (BMI) was calculated by the formula weight (kg)/ (height) ^2^ (m^2^). To determine the changes in fasting blood sugar and lipid concentration, at baseline and the end of the study, 5 cc blood samples were collected, and after the serum was extracted by high-speed centrifugation, the samples were immediately frozen at -70 °C until analysis. In this study FBS and lipid profile were defined as primary outcomes and dietary habits as secondary outcome. Serum concentrations of fasting blood sugar (FBS), triglycerides (TGs), high-density lipoprotein cholesterol (HDL-C), and total cholesterol (TC) were measured by enzymatic methods using commercial kits (Pars Azmoon, Tehran, Iran). The concentration of low-density lipoprotein cholesterol (LDL-C) was calculated based on the Friede-Wald formula.

To assess dietary habits, a food frequency questionnaire (147 items) was used at baseline and the end of the study. The validity and reliability of the questionnaire were evaluated in a previous study [33]. During the study, participants were asked to be on the usual diet and were monitored weekly to ensure the use of supplements. To assess compliance, data from eligible participants who consumed at least 90% of the supplements were entered into the analysis [34]. To facilitate the interpretation of dietary habit data, all items were divided into six food groups: cereals, milk and dairy products, meats, vegetables, fruits, and oils.

Based on the “body mass” variable [mean (SD)=-0.5 [1] in the intervention group and − 1.4 [1] in placebo in week 4) in the same study [35] and by considering power 90%, the confidence interval 95%, two-tailed test, finally, the sample size was obtained at least 26 subjects in each group. However, it was increased to 30 people due to the potential dropout during the follow-up period.

### Statistical analysis

For analysis, SPSS software (version 20; SPSS Inc., Chicago, IL) was used. The normality of the data was assessed by the Kolmogorov‒Smirnov test. The mean (standard deviation) was used to show numerical data, and categorical data were presented as frequencies (percentages). The baseline comparison of variables was performed by independent sample t tests and the chi-squared test (for quantitative variables and qualitative variables, respectively). The within-group changes in variables were analyzed by paired sample t-tests. The confounding factors included age, sex, and occupational and educational status. After adjusting for baseline measurements and confounding factors, we used the analysis of covariance (ANCOVA) test to evaluate the differences between groups. *P* values less than 0.05 were considered to indicate statistical significance.

## Results

**Fig. 1:**
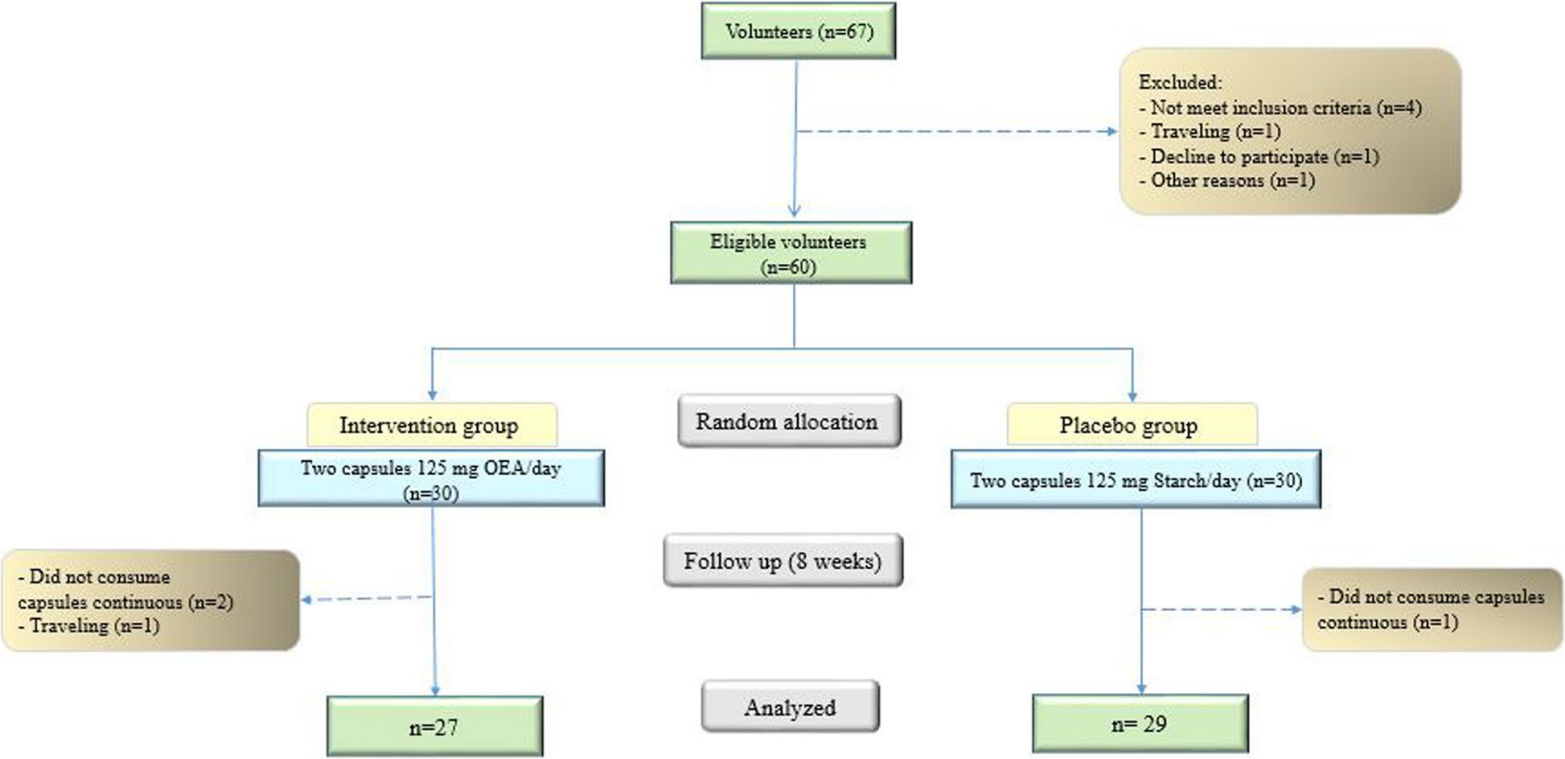
Flowchart of the study from baseline until the end of the study [15]

By considering inclusion and exclusion criteria among 67 volunteers, 60 obese people were entered into the study. At the end of the follow-up period, 27 participants in the intervention group and 29 in the placebo group completed the study. The physical activity of participants did not show a significant difference at the end of study compared baseline (data were not shown). The flowchart of the study is shown in Fig. 1 [20].

Approximately 45% and 35% of participants in the intervention and placebo groups, respectively, were males (*p* > 0.05). The mean (SD) BMI in the intervention group was 34.69 (2.40) kg/m^2,^ and that in the placebo group was 35.08 (2.87) kg/m^2^ (*p* > 0.05). Table 1 shows the demographic characteristics and biochemical data of the participants at baseline.

**Table 1:** The demographic characteristics of obese people at the onset of the study (*n* = 56)

Variables	Intervention group (*n* = 27)	Placebo group (*n* = 29)	*p*^a^
Age (y)^b^	37.37 (8.74)	38.13 (9.28)	0.752
Gender^c^			
Female	15 (55.6)	19 (65.5)	0.446
Male	12 (44.4)	10 (34.5)	
Education level^b^			
- Illiterate	13 (48.1)	15 (51.7)	0.010
- Diploma	3 (11.1)	11 (37.9)	
- Bachelor’s degree and above	11 (40.7.6)	3(10.3)	
Occupation^b^			
- Clerk	8 (29.6)	4 (13.8)	0.348
- Housewife	14 (51.9)	19 (65.5)	
- Worker	5 (18.5)	6 (20.7)	
Weight (kg)^b^	93.01 (13.22)	91.25 (13.66)	0.272
BMI (kg/m^2^)^b^	34.69 (2.40)	35.08 (2.87)	0.838
FBS (mg/dL)^b^	89.77 (14.93)	84.48 (16.79)	0.219
TC (mg/dL)^b^	173.96 (32.58)	178.48 (37.38)	0.633
TG (mg/dL)^b^	147.77 (77.12)	175.93 (74.02)	0.169
LDL-C (mg/dL)^b^	90.68 (32.65)	98.55 (31.44)	0.363
HDL (mg/dL)^b^	48.74 (10.54)	47.31 (7.87)	0.566

^a^Independent sample t-test/Chi^2^ test

^b^presented as the mean (SD)

^c^presented as frequency (percent)

At the end of the study, the TG concentration decreased significantly in the intervention group (mean (SD): 166.29 (70.01) mg/dL to 142.22 (48.05) mg/dL, *p* = 0.047). Changes in the placebo group were not significant (*p* > 0.05). According to the ANCOVA, the mean difference in TG between groups after adjusting for baseline values and demographic characteristics remained significant (*p* = 0.044). Changes in other biochemical parameters were not significant (*p* > 0.05). Table 2 shows the mean difference between FBS and lipid concentration in both groups after adjusting for baseline values and demographic characteristics.

**Table 2:** The effects of OEA supplementation on the FBS and lipid profile biomarkers in obese people (*n* = 56)

Variables	Intervention group (*n* = 27)	Placebo Group (*n* = 29)	Mean Diff (95% CI) *p*^b^
**FBS**			
Before	91.62 (18.94)	85.86 (20.18)	0.96 (-9.69 to 11.61) 0.857
After	90.07 (19.4)	91.03 (20.36)	
Mean Diff (95% CI) p ^a^	-1.55 (-6.10 to 2.99) 0.489	5.17 (-4.47 to 10.81) 0.071	
**TC**			
Before	173.96 (32.58)	178.48 (37.38)	-1.38 (-20.93 to 18.16) 0.888
After	170.59 (33.29)	169.20 (39.18)	
Mean Diff (95% CI) p ^a^	-3.37 (-10.86 to 4.11) 0.363	-9.27 (-20.17 to 1.62) 0.092	
**TG**			
Before	166.29 (70.01)	175.93 (74.00)	31.41 (0.56 to 62.09) 0.044
After	142.22 (48.05)	173.55 (64.85)	
Mean Diff (95% CI) p ^a^	-24.07 (-47.85 to -0.29) 0.047	-2.37 (-32.20 to -27.44) 0.871	
**LDL-C**			
Before	90.68 (32.65)	98.55 (31.44)	-2.93 (-19.51 to 13.63) 0.724
After	96.09 (31.41)	93.15 (30.43)	
Mean Diff (95% CI) p ^a^	5.40 (-7.39 to 18.21) 0.393	-5.39 (-15.57 to 4.79) 0.287	
**HDL-C**			
Before	48.74 (10.54)	47.31 (7.87)	0.08 (-5.69 to 5.85) 0.978
After	47.33 (12.10)	47.41 (9.30)	
Mean Diff (95% CI) p ^a^	-1.40 (-4.65 to 1.84) 0.382	0.10 (-2.75 to 2.96) 0.941	

Data were presented as Mean (SD)

^a^Paired t Test

^b^ANCOVA test (Adjusting on baseline values, age, gender, education, and occupation variables)

There was no significant difference between or within groups in terms of food groups. Table 3 shows the mean differences in food groups after adjusting for baseline values and demographic characteristics.

**Table 3:** The effects of OEA supplementation on the fasting blood glucose and lipid profile biomarkers in obese people (*n* = 56)

Variables	Intervention group (*n* = 27)	Placebo Group (*n* = 29)	Mean Diff (95% CI) *p*^b^
**Cereal**			
Before	81.25 (15.33)	88.24 (14.15)	6.45 (-3.93 to 16.84) 0.217
After	81.40 (18.83)	87.86 (19.85)	
Mean Diff (95% CI) P ^a^	0.14 (-3.71 to 4.01) 0.938	-0.37 (-5.48 to 4.73) 0.880	
**Dairy**			
Before	18.77 (8.24)	19.17 (7.45)	3.32 (-1.87 to 8.53) 0.205
After	19.22 (10.07)	22.55 (9.35)	
Mean Diff (95% CI) P ^a^	0.44 (1.08 to -1.97) 0.556	3.37 (1.78 to 497) < 0.001	
**Meat**			
Before	22.40 (11.60)	24.83 (10.91)	1.44 (-4.12 to 7.02) 0.605
After	21.51 (10.80)	22.96 (9.98)	
Mean Diff (95% CI) P ^a^	-0.88 (-1.14 to 2.91) 0.376	-1.86 (-3.88 to 0.15) 0.069	
**Fruit**			
Before	24.48 (7.80)	24.20 (6.28)	-1.98 (-7.39 to 3.41) 0.464
After	25.33 (10.82)	23.34 (9.33)	
Mean Diff (95% CI) P ^a^	0.85 (-1.50 to 3.21) 0.465	-0.86 (-3.36 to 1.64) 0.487	
**Vegetable**			
Before	34.59 (18.32)	31.37 (13.83)	-1.98 (-10.32 to 6.36) 0.636
After	35.74 (17.48)	33.75 (13.54)	
Mean Diff (95% CI) P ^a^	1.14 (-1.32 to 3.62) 0.349	2.37 (-1.01 to 5.77) 0.162	
**Oil**			
Before	31.92 (7.50)	34.62 (9.64)	4.78 (-1.69 to 11.26) 0.142
After	32.59 (10.83)	37.37 (13.14)	
Mean Diff (95% CI) P^a^	0.66 (-2.09 to 3.42) 0.624	2.75 (0.54 to 4.97) 0.017	

Data were presented as Mean (SD)

^a^Paired t Test

^b^ANCOVA test (Adjusting on baseline values, age, gender, education, and occupation variables)

## Discussion

Obesity, as the main outcome of the modern lifestyle, appears to be a sedentary lifestyle, improper diet, irregular meals, snacking, and overeating during break times [36]. Excess calories are stored in body tissues as triglycerides, commonly known as fat, and the adipocytes where triglycerides are stored are known as fat cells [37].

In this study, OEA significantly decreased the TG concentration and exerted no obvious effects on the other lipid profile parameters and FBS. In agreement with this study, *Tutunchi* et al. [28] showed that 12 weeks of supplementation with 250 mg OEA significantly decreased TG in obese subjects with NAFLD. However, in contrast to the present study, OEA significantly increased HDL-C. *Verma* et al., [38] in a retrospective investigation, showed that supplementation of 100 obese people with 200 mg OEA, 75 mg pantethine, and 10 mg valine for 3 months significantly decreased lipid biomarkers, including TG, TC, and LDL. A subgroup analysis of diabetic patients showed that OEA decreased FBS significantly at the end of the study. According to *Fu* et al., [39] treatment of rats with 5 mg/kg body weight OEA once daily for two weeks significantly decreased TG levels but had no obvious effect on glucose levels. In this study, OEA enhanced the gene expression of PPAR-α and other PPAR-α target genes, including liver fatty-acid binding protein (L-FABP), fatty-acid translocase (FAT/CD36), and uncoupling protein-2 (UCP-2). In a survey by *Chen* et al., [40] treatment of mice with 5 mg/kg/day OEA significantly decreased the accumulation of TG in the liver and inhibited the increase in the serum TG concentration. *Tovar* et al. [41] showed that treatment of 8 rats with OEA (10 mg/kg) for 15 days did not influence TG or LDL-C. Similarly, Guzman et al., [42] showed that treatment with 5 mg/kg b/w OEA for 4 weeks did not impact glucose uptake or glucose oxidation.

**Fig. 2:**
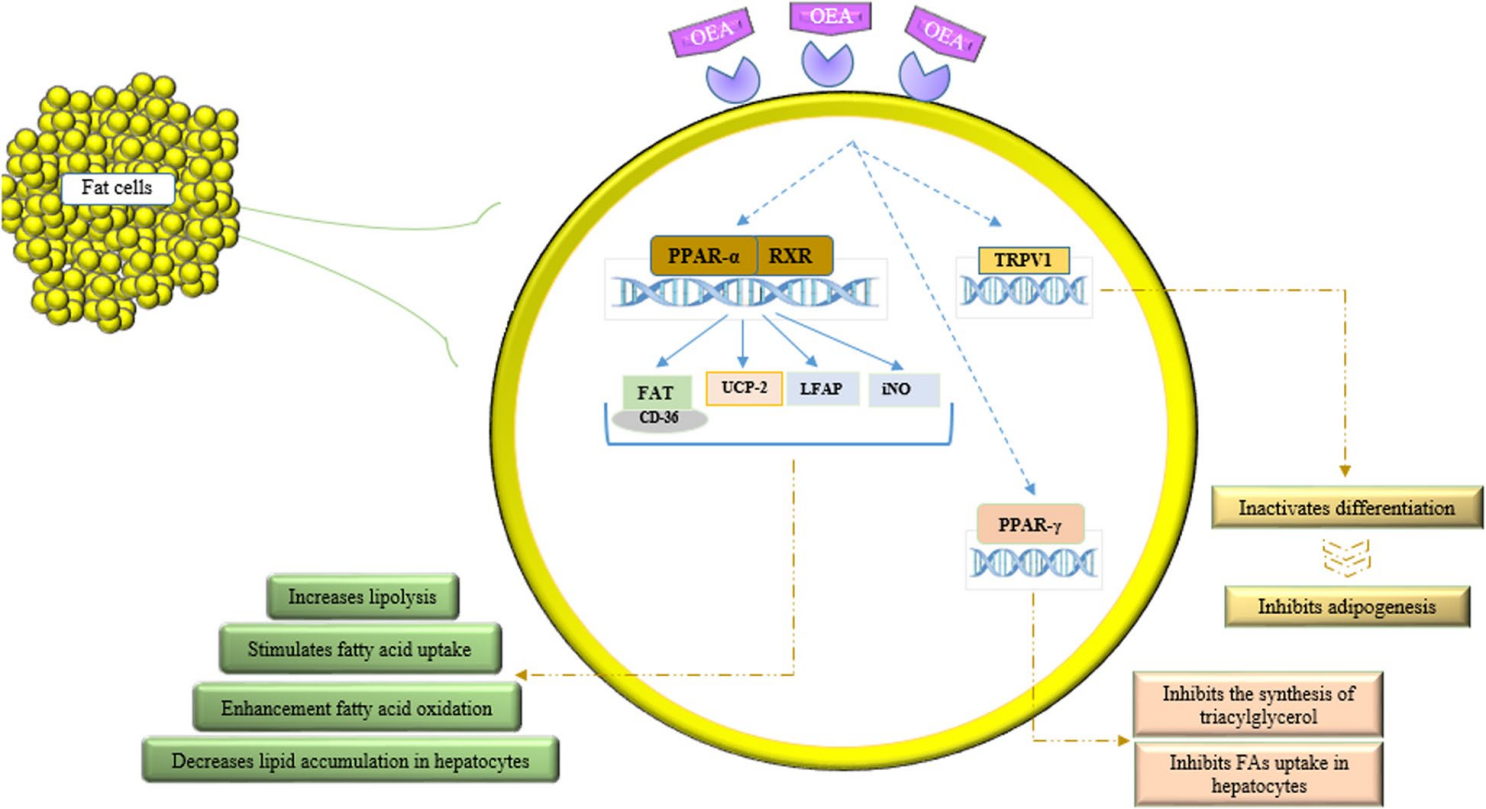
The potential mechanisms by which OEA affects TG levels

The exact mechanisms by which OEA modulates lipid profiles and glucose levels are not fully understood. However, one of the main functions of OEA is its involvement in lipid metabolism, stimulation of fatty acid uptake, decrease in lipid accumulation in hepatocytes, and enhancement of fatty acid oxidation and lipolysis. In addition to modulating lipid metabolism, OEA can relieve inflammation, energy, and glucose homeostasis and improve anthropometric measurements and feeding behaviors [19, 39, 40, 43–45]. OEA exerts these effects by increasing the expression of genes such as PPAR-α and fatty acid translocase (FAT)/CD36, which are involved in fat metabolism, lipolysis, and glucose hemostasis [46–48]. PPAR-α, a group of ligand-activated nuclear receptors, controls the transcription of genes involved in metabolism, such as fatty acid translocase, uncoupling protein-2, and liver fatty acid binding protein (LABP). In addition, it activates the transcription of proteins involved in lipid metabolism and inhibits the enzyme inducible nitric oxide (iNO) synthase, which stimulates feeding behaviors [44]. OEA increases the expression of the transient receptor potential vanilloid type 1 (TRPV1) gene in preadipocytes, which inhibits adipogenesis via inactivation of preadipocyte differentiation [2, 49]. Another mechanism is related to the activation of PPAR-γ by OEA, which inhibits the synthesis of triacylglycerol and fatty acid uptake in hepatocytes [50]. Figure 2 summarizes the potential mechanisms by which OEA regulates TG levels.

The conflicting results of the studies regarding the impact of OEA on lipid profiles and EBS may be related to differences in the target groups, study design, dose, and duration of the intervention. On the other hand, considering all the studies, a complete evaluation of the studied indicators has not been performed; hence, their complete interpretation is not possible; for that reason, conducting more studies in this field will be helpful.

In this study, OEA had no significant effect on the dietary habits of the participants. Previous studies confirmed the role of OEA in appetite and food intake by inhibiting feeding by prolonging meal intervals and decreasing meal frequency with no effect on meal size [20, 31, 51, 52]. According to the definition of dietary habits, it refers to the long-term intake of foods and is attributed to the feeding habits of individuals and maintains their life span [53], while dietary records mainly refer to the current intake of food groups in a short time [54]. Considering this, the lack of significant effects of OEA on dietary habits in this study may be due to the short duration of the intervention, while in previous studies, the effects of OEA on appetite, food intake, feeding behaviors, and anthropometric indicators were confirmed [19, 20]. To the best of our knowledge, this was the first study to assess the impact of OEA on dietary habits and lipid profiles along with FBS in obese people. Our study had several limitations, including a lack of assessment of the expression of genes involved in fat metabolism, such as TRPV1 and (FAT)/CD36, and a shorter duration of intervention.

In conclusion, the present study showed that supplementation of obese people with 250 mg OEA for 8 weeks significantly decreased the triglyceride concentration; however, no obvious effect on FBS, other lipid profile parameters, or dietary habits was detected. Future studies are needed to explore the mechanism of action of OEA in lipid modulation.

## Data Availability

All data generated or analyzed during this study are included in this published article

## Data availability

All data generated or analysed during this study are included in this published article.
